# Hereditary tyrosinaemia type 1 in the absence of succinylacetone: 4‐oxo 6‐hydroxyhepanoate (4OHHA), a putative diagnostic biomarker

**DOI:** 10.1002/jmd2.12436

**Published:** 2024-06-18

**Authors:** Preeya Rehsi, Karolina Witek, Erin Emmett, Rachel Carling, Charles Turner, Neil Dalton, Tim Hutchin, Nedim Hadzic, Anil Dhawan, Roshni Vara

**Affiliations:** ^1^ Department of Paediatric Inherited Metabolic Disease Evelina Children's Hospital London UK; ^2^ Biochemical Sciences, Synnovis, Guys & St Thomas' NHS Foundation Trust London UK; ^3^ Well Child Laboratory Evelina London Children's Hospital, Guy's and St Thomas' National Health Service Foundation Trust London UK; ^4^ Newborn Screening and Biochemical Genetics Birmingham Children's Hospital Birmingham UK; ^5^ Paediatric Liver, GI and Nutrition Centre and Mowat Labs King's College Hospital London UK

**Keywords:** 4‐oxo 6‐hydroxyhepanoate, emerging diagnostic biomarker, fumarylacetoacetate hydrolase, succinylacetone, tyrosinaemia type 1

## Abstract

Hereditary tyrosinemia type 1 (HT1) is a rare metabolic disease resulting in acute liver failure in early infancy, hypophosphataemic rickets, neurological crises, liver cirrhosis and risk of hepatocellular carcinoma later on in life. It is caused by the deficiency of the enzyme fumarylacetoacetate hydrolase which is involved in the terminal step of the catabolic pathway of tyrosine. Diagnosis is made through clinical suspicion supported by biochemical abnormalities that result from accumulation of upstream metabolites. Detection of succinylacetone (SA) in dried blood spot or urine remains pathognomonic, however it is not always detectable. Here we describe three cases of HT1 presenting with atypical biochemistry, where SA was not always detectable, highlighting the importance of an additional disease biomarker, 4‐oxo‐6‐hydroxyheptanoate.

**Synopsis**

Succinylacetone detection is pathognomonic of hereditary tyrosinemia type 1, but may not be detectable in all cases. An additional disease biomarker, 4‐oxo 6‐hydroxyheptanoate, detected in urine, may be of increasing importance in detecting mild or insidious disease.

## 1 INTRODUCTION

Hereditary tyrosinaemia type 1 (HT1) is an autosomal recessively inherited metabolic condition resulting from the deficiency of the enzyme fumarylacetoacetate hydrolase (FAH; EC 3.7.1.2) involved in the terminal step of the catabolic pathway of the amino acid tyrosine. This enzyme deficiency is caused by pathogenic variants in the *FAH* gene responsible for enzyme expression mainly in the liver and kidneys. In geographic areas without newborn screening, HT1 affects approximately one in 100 000 births. Certain parts of the world have higher incidences due to local founder effects, for example, Finland 1: 60000 and Quebec 1: 2000.^1^, ^2^ Despite many pathogenic variants identified in the *FAH* gene there does not appear to be a clear genotype/phenotype correlation.^3^, ^4^, ^5^

FAH deficiency results in the accumulation of upstream compounds including fumarylacetoacetate (FAA), maleylacetaceate (MAA) and their derivatives succinylacetone (SA; 4,6 dioxoheptanoic acid) and succinylacetoacetate (SAA; 3,5‐dioxooctanedioic acid), which exert their own pathogenic affects. The presentation of HT1 is variable and can be classified according to age. The acute form occurs before the age of 6 months typically presenting with acute liver failure (defined as international normalised prothrombin ratio (INR) >2.0 and jaundice with or without encephalopathy). Between 6 months and 1 year of age, this intermediate form presents with symptoms and signs of liver and renal disease: faltering growth, hypotonia, coagulopathy, hepatosplenomegaly and hypophosphataemic rickets. A chronic form of the disease presents after the first year of life with chronic liver disease, renal disease, rickets, cardiomyopathy, and a porphyria‐like crises. The liver is the principal organ affected in this condition with increased risk of early hepatocellular carcinoma (HCC) particularly if left untreated.

Significant morbidity and mortality are associated with untreated HT1, but the disease course has significantly improved since pharmacological treatment with Nitisinone was introduced in 1992 alongside dietary modifications.^6^ The biochemical rationale behind the use of Nitisinone is to block tyrosine degradation at an early step to prevent the production of toxic downstream metabolites such as MAA, FAA and SA. In combination with dietary restriction of tyrosine and phenylalanine, early initiation of treatment significantly reduces the risk of developing HCC. Renal tubular dysfunction resolves quickly, rapid improvement in liver function is seen, and progression of chronic liver disease is rare with good treatment adherence. Liver transplantation is now only reserved for patients with liver failure unresponsive to pharmacological treatment, or evidence of hepatic malignant changes.^7^

Diagnosis is based on a high index of clinical suspicion in patients with liver dysfunction (often with elevated transaminases and highly elevated alpha‐feto protein (AFP)), coagulopathy and renal Fanconi‐like syndrome. Diagnostic biochemical abnormalities include elevated plasma tyrosine (this may also be within normal range^8^), phenylalanine and methionine, and increased urinary 5‐aminolevulinic acid (5‐ALA). The latter results from secondary inhibition of 5‐ALA dehydratase by SA. The detection of SA in dried blood spot (DBS), plasma or urine is regarded pathognomonic of HT1. SA is a sensitive and specific marker for HT1 and its measurement in DBS using flow injection analysis tandem mass spectrometry (FIA‐MS/MS) has been used for newborn screening in several countries.^9^, ^10^, ^11^ Urine SA may be undetectable in mild cases and this is thought to be secondary to residual FAH enzyme activity in these patients.^12^

Here we describe three cases of HT1 presenting with atypical biochemistry, negative SA and identification of an additional disease biomarker, 4‐oxo‐6‐hydroxyheptanoate. (Table 1).

**TABLE 1:** Patient characteristics.

	Case 1	Case 2	Case 3
Gender	Female	Female	Female
Ethnicity	Pakistani	Arab	Arab
Consanguinity	Yes	Yes	Yes
Age at presentation	6 weeks	9 months	10 months
Presenting symptoms	Acute liver failure Seizures Hypoglycaemia Multi‐organ dysfunction	Cirrhotic liver disease	Hepatosplenomegaly Coagulopathy Elevated transaminases
Tyrosine (μmol/L)	763	N/A	179
Newborn DBS SA (μmol/L)	1.94	N/A	N/A
Repeat DBS SA (μmol/L)	N/A	0.47	Positive*
Urine SA (Y/N)	N	Y then N	Y
Urine 4‐oxo 6‐hydroxyheptanoate (Y/N)	Y	Y	Y
Skin cultured fibroblasts FAH activity U/g protein (Normal 1.07–3.76)	0.43	0.2	0.08
Genetics	*FAH* c.974C>T p.(Thr325Met)	*FAH* c.841C>A p.(Pro281Thr)	*FAH* c.841C>A p.(Pro281Thr)
Age of last follow up	3 months	16 years	2 years
Outcome	Deceased	Alive	Alive

Abbreviations: N, no; N/A, not available; Y, yes.

* Exact value not available.

## 2 CASE 1

A six‐week old female, born to consanguineous parents of south‐Asian origin, presented with acute liver failure, seizures, hypoglycaemia and multi‐organ dysfunction. At presentation plasma tyrosine was 1031 μmol/L (range 26–154 μmol/L), phenylalanine 763 μmol/L (range 34–110 μmol/L) and methionine 1139 μmol/L (range 10–53 μmol/L). The INR was elevated at 4.6 (normal <1.0). The serum AFP level was 64 996 IU/L. Urine organic acid analysis revealed marked increases in the excretion of 4‐hydroxyphenyl‐lactate, 4‐hydroxyphenyl‐pyruvate and n‐acetyl‐tyrosine with a mild increase in vanillyllactate (HMDB), reflecting the liver dysfunction. No SA was detected. Plasma SA measured by liquid chromatography tandem mass spectrometry (LC–MS/MS)^13^ was negative (<0.2 μmol/L). She underwent full diagnostic work up for acute liver failure and passed away aged 3 months without a confirmed diagnosis. Subsequently, trio whole genome sequencing (WGS) analysis identified a homozygous *FAH* missense variant NM_000137.4:c.974C>T p.(Thr325Met), previously reported in compound heterozygote state,^14^, ^15^ suggesting a diagnosis of HT1. Retrospective review of the urine organic acid chromatogram confirmed that whilst there was no detectable SA, 4‐oxo 6‐hydroxyheptanoate was present. To obtain further biochemical evidence, the original newborn screening specimen, a DBS sample collected on day 5 of life was retrieved with parental consent. Subsequent analysis confirmed the presence of SA and tyrosine (1.94 μmol/L and 679 μmol/L respectively). Further diagnostic confirmation was provided from cultured skin fibroblasts which revealed low FAH activity 0.43 U/g protein (1.07–3.76) consistent with a diagnosis of HT1.

## 3 CASES 2 AND 3

A nine‐month‐old female (Case 2), born to consanguineous parents of Arab origin, presented with cirrhotic liver disease. During work up for liver transplantation tests revealed a trace of SA in urine which was negative on repeated samples. The plasma amino acids were all within normal range and the serum AFP was 2694 IU/L. She underwent extensive investigation for chronic liver disease and remained stable with a compensated cryptogenic chronic liver disease until the last follow up aged 16 years. She has normal liver function tests and serum AFP persistently elevated at 25 IU/L. Liver imaging could not detect any focal nodules and the liver parenchyma is heterogeneous with a mildly enlarged spleen at the upper limit of normal.

Case 3, the younger sibling of Case 2 presented at 10 months of age with hepatosplenomegaly, coagulopathy, elevated transaminases and serum AFP 32000 IU/L. Plasma tyrosine levels were marginally elevated at 179 μmol/L. Urine organic acid analysis revealed a marginal increase in the excretion of SA and 4‐oxo 6‐hydroxyheptanoate suggestive of HT1. A DBS specimen was positive for SA supporting the diagnosis of HT1 suspected clinically. Treatment with Nitisinone (NTBC) was commenced along with dietary intervention. A genetic cholestasis panel revealed homozygosity for a variant of unknown significance in *FAH* c.841C>A p.(Pro281Thr) with in silico predictors of pathogenicity suggesting that it is damaging. Skin fibroblast culture was undertaken and enzymology showed FAH deficiency (FAH activity 0.08 U/g protein (range 1.07–3.76)).

In view of the findings in Case 3, Case 2 was reviewed and new specimen obtained. The DBS specimen was positive for SA (0.47 μmol/L) and a marginal increase in SA and 4‐oxo‐6‐hydroxyheptanoate were detected in the fresh urine. Cultured fibroblast enzymology revealed low FAH activity 0.2 U/g protein (1.07–3.76) consistent with a diagnosis of HT1. Treatment was commenced on low dose Nitisinone with a view to obtain undetectable dried blood spot SA, but was refused initially due to concerns over adverse effects including abdominal pain and dietary intervention. Clinical surveillance using serum AFP and liver imaging is ongoing.

## 4 URINE ORGANIC ACID ANALYSIS BY GAS CHROMATOGRAPHY MASS SPECTROMETRY (GC–MS)

**FIGURE 1:**
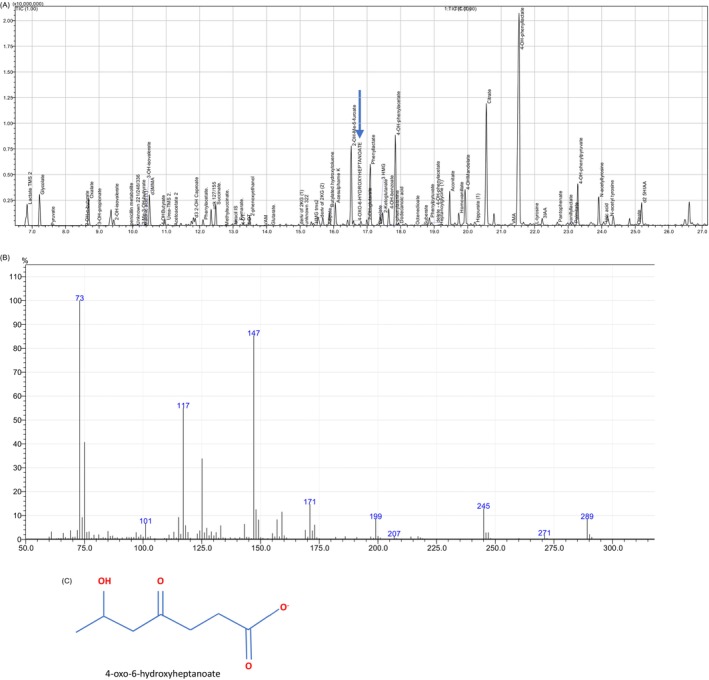
(A) Total ion current (TIC) chromatogram from case 1 showing elution of 4‐oxo‐6‐hyroxyheptanoate at 16.6 min (arrow). (B) Non‐oximated spectrum of 4‐oxo‐6‐hydroxyheptanoate (diTMS). (C) Structure of 4‐oxo‐6‐hydroxyheptanoate (C_7_H_12_O_4_).

Following acidification of urine, organic acids were extracted by sequential liquid–liquid extraction (ethylacetate/diethyl ether) and the solvent extract was evaporated under nitrogen prior to reconstitution with pyridine and derivatisation with BSTFA/1%TMCS. Succinylacetone was extracted using non‐oximated organic acid sample preparation protocol and analysis was performed on a Shimadzu QP 2010 ULTRA GC (Shimadzu, Milton Keynes UK) coupled to a non‐polar (HP‐5 ms Ultra) fused silica capillary column (30 m × 0.25 × 0.25 μm) (Agilent, Didcot UK). Data were acquired in full scan mode from m/z 50–550. For cases 2 and 3, extracted ion chromatogram (XIC) m/z used for identification of SA (and SA stable isotope 3,4,5,6,7‐13C5) were 157 (161), 269 (174) and 287 (292). SA stable isotope was present at 2 μmol/L to confirm adequate SA detection at this low concentration. The initial sample from Case 1 was analysed 10 years prior to cases 2 and 3 using oximated sample preparation protocol and no SA stable isotope was in use at that time (Figure 1). Extracted ion chromatogram (XIC) m/z used for identification of 4‐oxo‐6‐hydroxyheptanoate were 287/199/171.

The creatinine concentrations of the samples were 1.6, 24.5 and 1.3 μmol/mmol Cr for Cases 1, 2 and 3 respectively and the limit of quantitation of succinylacetone is 0.6 μmol/mmol creatinine.

## 5 DISCUSSION

We present 3 cases of HT1 which demonstrate the variable clinical phenotype, the potential for urinary SA to be undetectable and the clinical utility of an additional disease biomarker, 4‐oxo‐6‐hydroxyhepatanoate. Despite early onset and severe disease, Case 1 had persistently undetectable SA in urine and plasma at presentation. However post‐hoc review of the newborn screening specimen showed elevated SA supporting the use of SA in newborn screening which has already been employed by several countries.^9^, ^11^, ^14^, ^15^, ^16^, ^17^ The use of DBS SA is more convenient than plasma and urine and further strengthens the current recommendation for newborn screening in the United Kingdom using this method. The exact cause of undetectable SA in plasma or urine during the acute presentation in Case 1 remains uncertain. Previous cases of HT1 have been reported with undetectable SA levels (i.e., ‘silent tyrosinaemia’) and associated with a presumed milder phenotype suggesting that residual enzyme activity causes the biochemical variability in diagnosis.^12^, ^18^ Another hypothesis is that there is impairment of the tyrosine catabolic pathway due to prolonged liver failure; enzyme synthesis ceases thereby reducing the production of metabolites that we would otherwise detect earlier in the acute illness. This may also potentially explain the lack of detection of SA in Case 2 at initial presentation with cirrhotic liver disease.

Almost all laboratories measure SA, SAA and 4‐oxo‐6‐hydroxyheptanoate, by GC–MS, as part of a urinary organic acid profile which detects over 150 compounds in a single analysis. As such, the methods is not optimised for the detection of these metabolites, measurement is qualitative only and can be challenging, especially at low levels. There are widely acknowledged but not necessarily evidence‐based strategies to improve the confidence with which SA is detected; oximation of the sample, use of an alternative derivatisation agent and detection via a single ion monitoring rather than full scan. Irrespective of which strategy is adopted, addition of stable isotope labelled SA internal standard (IS) at low concentration is critical to monitor the ability of the method to detect low levels of SA. As the specimen from Case 1 was analysed prior to the inclusion of SA IS in the laboratory's method it is therefore possible that SA was undetectable due to losses during the sample preparation process. However, this could have massive implications for the clinical decision‐making process, as clinicians would not normally question reported negative SA.

The DBS method is set up to detect SA and uses acidification to release SA from the Schiffs bases which form between SA and amine groups. Plasma may be less sensitive because some SA will be lost due to these reactions when the cells are removed, particularly if there is a delay in separation, even if the sample is subsequently acidified. In our patients the plasma and DBS samples were not taken at the same time so direct comparisons between sensitivities using our methods could not be made. Urine would be expected to give similar diagnostic sensitivity to DBS, however conversion to 4‐oxo‐6‐hydroxyheptanoic acid may reduce the amount of SA appearing in urine, and urine dilution may affect the efficiency of extraction and derivatisation for GCMS.

**FIGURE 2:**
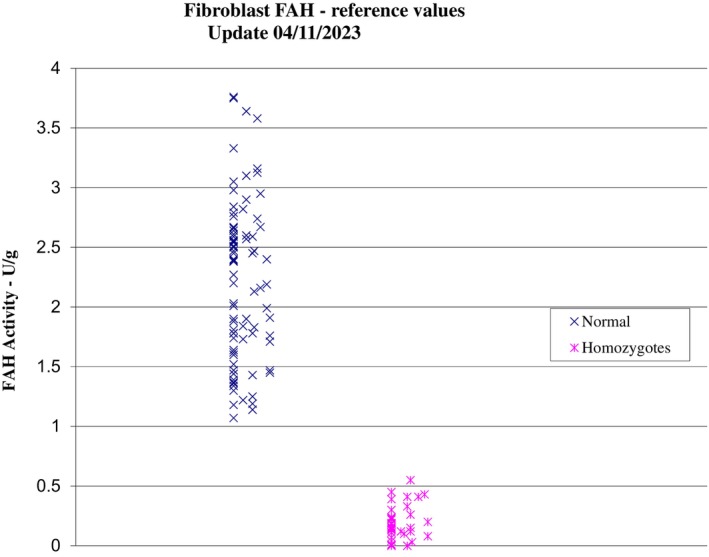
FAH fibroblast activity (unpublished data courtesy of T Hutchin).

The detection of 4‐oxo 6‐hydroxyheptanoate in urine could be a helpful diagnostic marker in the acute and chronic presentation of HT1. The compound, 4‐oxo 6‐hydroxyheptanoate is a derivative of SA^19^ so its detection in the urine of patients with HT1 may suggest that it is a sensitive marker, particularly where SA is not detected. In HT1 FAA accumulates and then is reduced to succinyl‐acetoacetate which is decarboxylated to SA and then reduced to 4‐oxo‐6‐hydroxyheptanoate. Case 3 demonstrates the detection of this metabolite also in the presence of a marginal increase in SA, with a typical phenotype. The *FAH* variant detected proved pathogenic through in silico studies and was further supported by low *FAH* activity in the cultured fibroblasts. NTBC treatment was commenced with dietary modification resulting in undetectable SA levels measured in DBS and clinical improvement. Case 2, sharing the same variant as Case 3, presented in infancy but showed a normalisation of liver function without treatment for several years. Functional studies showed a very low *FAH* activity in this case and why she remained stable for 16 years remains unclear. Fibroblast *FAH* activity seen in all three cases is similar to those reported in patients presenting with classical HT1 who had detectable SA. Correlation between liver and fibroblast FAH activity is known and diagnosis using fibroblasts, a more easily accessible tissue, is established in clinical practice.^20^ Unpublished data (personal communication T Hutchin, see Figure 2) has also shown mean fibroblast FAH activity 0.184 U/g (range, 0–0.55) in HT1 patients, in comparison to control FAH activity 2.12 U/g (range, 1.07–3.76). Further clinical and biochemical information is needed to make a direct comparison, but nevertheless this suggests that the enzyme activity may not always correlate with the presence of conventional diagnostic metabolites in HT1.

Despite several pathogenic disease variants seen in HT1 a clear genotype/phenotype correlation has not been identified. The missense variant found in Case 1 has been described in few cases in the compound heterozygous state,^21^, ^22^ suggesting uncertainty as to its individual pathogenicity. However, our case clearly demonstrates its pathogenicity in the homozygous state with clinical, biochemical, and functional studies in keeping with early severe onset of the disease. The variant in Cases 2 and 3, *FAH* c.841C>A p.(Pro281Thr), is currently classified as a variant of uncertain significance, although in silico analysis suggests pathogenicity and functional studies were conclusive. Our cases further support its pathogenicity and demonstrate association with both an acute and chronic disease course with an intrafamilial variability. It is clearly extremely important to continue reporting these phenotypes to the centralised genetic databases.

In summary these three cases represent the diagnostic challenges that can be experienced in HT1 both clinically and biochemically. The pathognomonic biomarker SA can be undetectable in both urine and plasma in severe and intermediate onset of disease, with no obvious correlation to the enzyme activity. Such cases can therefore be potentially missed using standard diagnostic methods, in particular in patients with a more insidious clinical presentation. Therefore, where clinical suspicion arises, even in the absence of SA, it is important that the organic acid chromatogram is carefully inspected for the presence of the additional biomarker 4‐oxo 6‐hydroxyheptanote. At present, there is insufficient data to use this compound alone as a diagnostic marker and further evidence should be sought from additional genetic testing and functional studies. Further validation of this biomarker including its sensitivity and specificity is necessary.

## 6 CONCLUSION

We highlight potential use of the additional analysis of urinary 4‐oxo 6‐hydroxyhepanoate in suspected cases of HT1 where initial biochemical tests (plasma tyrosine levels/ plasma or urine SA) may not be diagnostic. Further diagnostic evidence should be sought from functional studies and genetic testing when 4‐oxo 6‐hydroxyhepanoate is detected in isolation. In conjunction with genetic studies, this metabolite may be of increasing importance in the identification of milder or more insidious forms of HT1 or where SA is not clearly detectable.

## AUTHOR CONTRIBUTIONS

As first author PR contributed to the design, acquisition of data, analysis and drafting of the work. Co‐authors KW, EE, RC, TH, CT and ND provided interpretation and expertise in the biochemical and laboratory aspects of the work. Co‐authors NH and AD participated in the analysis and expertise of the hepatology aspects of the work. Senior author and guarantor RV participated in the conception, design, acquisition of data, critical review and revision of the work. All authors reviewed the work and provided final approval of the version to be published.

## FUNDING INFORMATION

The authors received no financial support for the research or authorship of this article.

## CONFLICT OF INTEREST STATEMENT

Preeya Rehsi, Karolina Witek, Erin Emmett, Rachel Carling, Charles Turner, Neil Dalton, Tim Hutchin, Nedim Hadzic, Anil Dhawan and Roshni Vara declare they have no conflict of interest.

## ETHICS STATEMENT

Ethics approval was not required for this case report.

## INFORMED CONSENT

All procedures followed were in accordance with the ethical standards of the responsible committee on human experimentation (institutional and national) and with the Helsinki Declaration of 1975, as revised in 2000 (5). Informed consent was requested from all patients for being included in the study and currently awaited.

## ANIMAL RIGHTS

This article does not contain any studies with human or animal subjects performed by the any of the authors.

## Data Availability

The participants of this study did not given written consent for their data to be shared publicaly. Sharing anonymised data can be discussed upon request from the authors.
